# Associations Between Growth Differentiating Factor‐15 and Frailty in Older Adults From the MAPT Study

**DOI:** 10.1002/jcsm.70182

**Published:** 2026-01-20

**Authors:** Juan Luis Sánchez‐Sánchez, Yves Rolland, Alexandre Lucas, Sophie Guyonnet, Bruno Vellas, Philipe de Souto Barreto, for the MAPT/DSA Group

**Affiliations:** ^1^ IHU HealthAge Toulouse France; ^2^ Institut du Vieillissement, Gérontopôle de Toulouse, Centre Hospitalo‐Universitaire de Toulouse Toulouse France; ^3^ CERPOP UMR 1295 University of Toulouse III, Inserm, UPS Toulouse France; ^4^ Institut National de la Santé et de la Recherche Médicale (INSERM), UMR 1297 Institute of Metabolic and Cardiovascular Diseases Toulouse France

**Keywords:** ageing, biomarkers, frailty, growth differentiation factor 15

## Abstract

**Background:**

Frailty is a prevalent syndrome in older adults and is associated with increased vulnerability to adverse health outcomes. Growth differentiation factor 15 (GDF‐15), a cytokine involved in mitochondrial dysfunction and inflammation, has been proposed as a potential biomarker for age‐related conditions. Evidence on the association between GDF‐15 and frailty in older adults is limited. This study explores the relationship between plasma GDF‐15 levels and frailty onset in community‐dwelling older adults.

**Methods:**

A secondary analysis was performed on 1096 participants (mean age = 75.2 ± 4.5 years; 64.5% women) from the Multidomain Alzheimer Prevention Trial (MAPT). Plasma GDF‐15 levels were measured at year 1. Frailty was assessed using the Fried phenotype. Logistic regression was used to examine cross‐sectional associations between GDF‐15 and frailty, while mixed effects logistic regression or Cox proportional hazards models assessed longitudinal associations over a 4‐year follow‐up.

**Results:**

Higher plasma GDF‐15 levels (both as continuous and categorical) were cross‐sectionally associated with frailty (high vs. low GDF‐15: OR = 3.56, 95% CI = 1.58–8.03). Longitudinally, very high GDF‐15 levels predicted an increased risk of incident frailty (HR = 1.69, 95% CI = 1.03–2.78).

**Conclusions:**

Elevated plasma GDF‐15 levels were associated with frailty in older adults, suggesting its potential as a biomarker for increased vulnerability and an indicator of increased risk over time. Our results support a pleiotropic role of GDF‐15, with low physiological levels not contributing to frailty development.

## 1 Introduction

Frailty is a syndrome associated with multiple causes, characterized by a reduction in the resistance to endogenous and exogenous stressors, leading to increased an individual's vulnerability [1]. This clinical condition is related to negative health outcomes, such as falls, fractures, reduced activities of daily living, disability, hospitalizations and mortality [2].

Despite the fact that frailty can be relatively easily assessed and identified in clinical settings and the community, the incorporation of easy‐to‐measure and reliable molecular biomarkers has the potential to provide better insights on its pathophysiology, to assist in the identification of potential therapeutic targets, improve the accuracy of diagnosis, stratify severity and understand which individuals might benefit the most from interventions [3, 4]. Although efforts have been performed in the incorporation of molecular biomarkers of frailty, no single predictive ones have been incorporated due to the lack of reproducibility [5, 6].

Growth differentiation factor 15 (GDF‐15), also known as macrophage inhibitory cytokine 1, is a cell stress‐responsive cytokine, member of the TFG‐β superfamily expressed in multiple tissues and organs [7]. It binds to the glial‐cell derived neurotrophic factor family receptor (GFRAL) in the hindbrain [8]. A pleiotropic nature of GDF‐15 has been described, with physiological levels playing a role in effective adaptation and appetite regulation [9]. On the other hand, high levels of the cytokine have been associated with various conditions, including chronic inflammation [10], cancer [11], cardiovascular [12] and kidney and liver diseases [13]. Both local expression and blood levels of GDF‐15 have been linked to mitochondrial dysfunction [14], anorexia [15], malnutrition [16] and muscle wasting [17], core pathophysiological contributors to frailty. Given the tight connections between increased GDF‐15 and both chronological [18] and biological [19] ageing, age‐related conditions [20], physical and cognitive function [21] and late‐life adverse outcomes [22], GDF‐15 has been suggested as a potential transversal biomarker in the Geroscience field. The latter is an interdisciplinary field that explores the relationship between ageing and chronic disease [23].

However, evidence on the association of circulating GDF‐15 and frailty is scarce, fragmented and restricted to nonanimal models or disease‐specific populations [24, 25, 26, 27], limiting our understanding of the role of the marker to identify individuals at risk of adverse events.

This study, relying on a relatively large sample size, aims to explore the prospective associations between plasma levels of GDF‐15 and frailty in a sample of community‐dwelling older adults. We hypothesize that higher baseline levels of GDF‐15 in plasma will be present among individuals with frailty and will associate with a heightened risk of presenting frailty over the 4‐year follow‐up.

## 2 Methods

### 2.1 Study Design and Population

This is a secondary observational analysis of the Multidomain Alzheimer Prevention Trial Study (MAPT, ClinicalTrials.gov [NCT00672685]), a multicentre, placebo‐controlled randomized clinical trial with community‐dwelling older adults in France and Monaco. The main objective of the MAPT study was to investigate the effect of a multi‐domain intervention (consisting of nutritional advice, physical activity recommendations and cognitive training) combined with omega‐3 PUFA supplementation on the cognitive health of older adults at risk of dementia. The 3‐year experimental phase was followed by a 2‐year observational phase. Participant inclusion started in May 2008 and ended in February 2011, whereas follow‐up ended in April 2016.

The detailed description of the MAPT study can be found elsewhere [28, 29]. Briefly, the eligibility criteria comprised: age ≥ 70 years, absence of major neurocognitive disorders, Mini‐Mental State Examination ≥ 24, and presenting one of the following: spontaneous memory concern, inability to perform at least one instrumental activity of daily living, or slow usual‐pace walking speed (< 0.8 m/s). Participants were excluded if they declared the use of ω‐3 PUFA during the prior 6 months. The population of the present study is composed of individuals with data on plasma GDF‐15 levels and frailty (*n* = 959, 87.5% of the sample) at the MAPT year 1 visit (when GDF‐15 was measured) as the baseline.

The whole study was approved by the local Ethical Committee (CPP SOOM II) and was authorized by the French Health Authority. Written informed consent was obtained from all participants. The study is described in detail in previous works. The present study followed the Strengthening the Reporting of Observational Studies in Epidemiology (STROBE) guideline.

### 2.2 Measures

#### 2.2.1 GDF‐15 Assessment

Nonfasting blood samples were collected at the 1‐year visit and were stored at −80°C. Plasma levels of GDF‐15 were assessed using the fully automated immunoassay platform Ella (Bio‐Techne, San Jose, CA, USA) as described elsewhere (19). Plasma GDF‐15 levels were quantified by a single disposable microfluidic SimplePlex cartridge using the fully automated immunoassay platform, Ella (ProteinSimple/Bio‐techne, San José, CA). Plasma samples were thawed on ice and diluted 1:4 in a sample diluent (SD 13) and loaded into cartridges with relevant high and low control concentrates. Each protein channel contains three analyte‐specific glass nanoreactors, which allow for each plasma sample to be run in triplicates for target protein samples. Cartridges include a built‐in lot‐specific standard curve for defined GDF15 protein. All steps in the procedure were run automatically by the instrument with no user activity. The obtained protein concentrations were calculated by the internal instrument software and displayed in pg/mL.

#### 2.2.2 Frailty and WL Outcomes

Frailty was assessed according to the Fried frailty phenotype, which is based on five components: (1) weakness (poor handgrip strength measured by a handheld dynamometer with sex‐specific and body mass index (BMI)–specific cut‐offs); (2) slowness (4‐m usual gait speed with cut‐offs established for men and women, according to height); (3) involuntary weight loss (self‐reporting > 4.5 kg of weight loss in the prior year); (4) exhaustion (according to two items of the Centre for Epidemiologic Studies depression scale); (5) low physical activity assessed by the 15‐item Minnesota Leisure Time Activity questionnaire (< 383 kcal/week in men and < 270 kcal/week in women during the prior 2 weeks).

Presence of frailty was defined as meeting ≥ 3 frailty criteria, pre‐frailty as presenting one or two criteria, and robustness as the absence of frailty criteria. Two frailty‐related endpoints were explored longitudinally. First, frailty evolution was captured by the changes in the frailty status along follow‐up. Second, incident frailty was defined as the onset of frailty from pre‐frailty/robustness at baseline in 865 subjects (median follow‐up, interquartile range = 2.93 ± 1.33).

#### 2.2.3 Confounders

Potential confounders consisted of age, sex, body mass index (BMI; kg/m^2^), MAPT group allocation, education (no diploma or primary school certificate, secondary education, high school diploma, university level) and plasma interleukin‐6 (IL‐6). The latter was included given its strong associations with frailty and as a marker of disease [30, 31]. We also included the number of comorbidities (among cardiovascular diseases, chronic respiratory disease, cancer, type 2 diabetes, depression and dementia). All confounders were measured at the 1‐year MAPT visit.

#### 2.2.4 Statistical Analysis

For descriptive purposes, subjects were split into two GDF‐15 groups according to the cut‐point proposed by Groarke et al. (plasma GDF‐15 ≥ 1500 pg/mL) to define individuals with high GDF‐15 [32]. Continuous variables are expressed as mean ± standard deviations and compared according to GDF‐15 status by means of the Student's *t* test. Categorical variables are expressed as frequencies and percentages, and compared by means of the *χ* ^2^ test.

The odds of presenting with a greater number of frailty criteria at baseline as a function of plasma GDF‐15 levels (either continuous or categorical) were explored by means of multivariate ordered logistic regression. The association between baseline GDF‐15 and the odds of presenting frailty at baseline was explored by logistic regression. The associations with the annual evolution in the number of present frailty criteria were explored by means of multilevel‐effects ordered logistic regression (MLEOL), with random intercept and slope for each participant. The MLEOL included as fixed effects the baseline plasma levels of GDF‐15 and time, their interaction and potential confounders. Cox's proportional hazards models were used to investigate associations of plasma GDF‐15 levels and frailty incidence. Time to first event was calculated for each outcome. Participants without the event were censored at their last follow‐up visit.

Estimates were computed as a function of GDF‐15 increases as continuous (1‐standard deviation increase), as high versus low based on the 1500 pg/L cut‐point, and quartiles (Q1 as reference). We used four different covariate structures across contrasts: age and sex (Model 1); model 1 plus BMI, educational level, MAPT allocation group (Model 2); model 2 plus IL‐6 (Model 3); and Model 3 plus the number of comorbidities (Model 4). We performed exploratory sub‐analyses stratified by sex and age groups (≤ 75 years vs. > 75 years), and with alternative inflammatory biomarkers as confounders (C‐reactive protein, tumour necrosis factor receptor‐1 and monocyte chemoattractant protein‐1).

## 3 Results

### 3.1 Baseline Characteristics and GDF‐15 Distribution

Table 1 shows the characteristics of the 1096 individuals with plasma GDF‐15 assessed at baseline (65.3% of the MAPT whole sample). Baseline characteristics by sex are displayed in Table S1. A comparison of the characteristics of MAPT participants included in present analyses and those not included at the MAPT year 1 visit (when both GDF‐15 and frailty were first assessed) are displayed in Table S2. Mean GDF‐15 was 1127.42 ± 511.69 pg/mL (coefficient of variation: 2.08% ± 1.19%). 169 (15.42%) of individuals were classified as having GDF‐15 levels ≥ 1500 pg/mL. Intra‐assay and interassay coefficient of variation were 12.6 ± 1.11%, and 2.07%1.19%, respectively. Subjects with high GDF‐15 were significantly older, more often men, were frailer, with higher odds of presenting the weight loss, low PA, fatigue and low gait speed frailty criteria, compared with the low GDF‐15 group.

**TABLE 1:** Baseline characteristics of the study population according to GDF‐15 status.

Variables	Total	Low GDF‐15	High GDF‐15
*N* (%)	1096	927 (84.58)	169 (15.42)
Age	75.30 ± 4.37	74.81 ± 4.05	78.00 ± 5.01
Female sex	613 (63.92%)	553 (67.77%)	60 (41.96%)***
MAPT group			
Multidomain intervention + omega‐3	274 (25.00%)	238 (25.67%)	36 (20.28%)
Omega‐3	267 (24.36%)	215 (23.19%)	52 (31.47%)
Multidomain intervention	276 (25.18%)	233 (25.13%)	43 (27.27%)
Placebo	279 (25.46%)	241 (26.00%)	38 (20.98%)
Body mass index (kg/m^2^)	26.22 ± 4.06	26.14 ± 4.01	26.66 ± 4.29
Education			
No diploma or primary school certificate	215 (22.73%)	185 (23.04%)	30 (20.97%)
Secondary education	320 (33.83%)	263 (32.75%)	57(39.86%)
High school diploma	146 (15.43%)	125 (15.57%)	21 (14.69%)
University level	265 (28.01%)	230 (28.64%)	35 (24.48%)
Fried frailty phenotype			
Robust	507 (52.87%)	453 (5.51%)	54 (37.76%)***
Pre‐frail	420 (43.80%)	344 (42.16%)	76 (53.15%)
Frail	32 (3.34%)	19 (2.33%)	13 (9.09%)
Fried frailty phenotype criteria			
Weight loss	19 (1.98%)	9 (1.10%)	10 (6.99%)***
Weakness	175 (18.25%)	143 (17.52%)	32 (22.38%)
Low physical activity	131 (13.66%)	100 (12.25%)	31 (21.68%)**
Fatigue	175 (18.25%)	143 (17.52%)	32 (22.38%)
Low gait speed	40 (4.17%)	24 (2.94%)	16 (11.19%)***
GDF‐15 (pg/mL)	1127.42 ± 511.69	960.70 ± 253.94	2041.91 ± 597.07***
IL‐6 (pg/mL)	3.89 ± 12.19	3.67 ± 13.11	5.10 ± 4.58

Abbreviations: GDF‐15, growth differentiation factor 15; IL‐6: interleukin 6.

* *p* < 0.05.

** *p* < 0.005.

*** *p* < 0.001.

### 3.2 Cross‐Sectional Associations With Frailty

A total of 959 individuals had data on GDF‐15 and frailty levels at baseline. In the ordinal logistic regression analyses, plasma baseline GDF‐15 levels were associated with higher odds of presenting 1 frailty criterion both when GDF‐15 was computed as continuous (per 1‐SD increase: odds ratio, OR = 1.22, 95% confidence interval, CI = 1.05–1.41) or dichotomous (≥ 1500 pg/mL vs. <1500 pg/mL: OR = 1.78, 95% CI = 1.24–2.56) across all adjustment models (Table 2). In quartile‐based analyses taking Q1 as the reference, the significant association was restricted to the Q4 (Q4 vs. Q1: OR = 1.71, 95% CI = 1.15–2.56). When frailty (≥ 3 frailty criteria) was used as the outcome, similar results were found in fully adjusted models, with higher GDF‐15 (higher than the median, according to quartile‐based results) being significantly associated with greater odds of frailty (Table 3). The absolute risk difference of Q3 and Q4 with respect to Q1 were 3.4% and 7.1%.

**TABLE 2:** Ordinal logistic regression examining cross‐sectional associations between plasma GDF‐15 and frailty status (odds of presenting 1 frailty trait more at baseline).

Plasma GDF‐15	Model 1^a^ (*N* = 959)			Model 2^b^ (*N* = 946)			Model 3^c^ (*N* = 945)					
	OR	95% CI	*p*	*p*	95% CI	*p*	OR	95% CI	*p*	Model 4^d^ (*N* = 945)		
Threshold 1: Cut‐off												
Low GDF‐15 (< 1500 pg/mL)	Ref.			Ref.			Ref.					
High GDF‐15 (≥ 1500 pg/mL)	**1.95**	**1.33–2.86**	**0.001**	**1.85**	**1.26–2.72**	**0.002**	**1.78**	**1.24–2.56**	**0.002**	**1.49**	**1.03–2.15**	**0.033**
Threshold 2: Quartiles												
GDF‐15 Q1 (≤ 799 pg/mL)												
GDF‐15 Q2 (> 799.0, ≤ 1009.0 pg/mL)	1.10	0.76–1.59	0.606	1.04	0.72–1.51	0.837	1.03	0.71–1.50	0.857	0.95	0.65–1.40	0.795
GDF‐15 Q3 (> 1009.0, ≤ 1312.0 pg/mL)	1.26	0.87–1.82	0.223	1.21	0.83–1.76	0.330	1.19	0.82–1.74	0.360	1.14	0.78–1.68	0.504
GDF‐15 Q4 (> 1312.0 pg/mL)	**1.90**	**1.28–2.81**	**0.001**	**1.77**	**1.18–2.63**	**0.005**	**1.71**	**1.15–2.56**	**0.009**	**1.56**	**1.03–2.35**	**0.034**
GDF‐15 as continuous												
1‐SD increase	**1.27**	**1.10–1.47**	**0.001**	**1.23**	**1.07–1.43**	**0.005**	**1.22**	**1.05–1.41**	**0.008**	**1.17**	**1.01–1.36**	**0.034**

*Note:* Significant associations in bold.

Abbreviations: CI, confidence interval; GDF‐15, growth differentiation factor 15; OR, odds ratio of increasing frailty severity over time compared with the reference group.

^a^ Adjusted by age and sex only.

^b^ Adjusted by age, sex, body mass index, MAPT group and education.

^c^ Adjusted by age, sex, body mass index, MAPT group, education and plasma IL6.

^d^ Adjusted by age, sex, body mass index, MAPT group, education, plasma IL6 and number of comorbidities.

**TABLE 3:** Logistic regression examining cross‐sectional associations between plasma GDF‐15 and frailty (presenting > 3 criteria at baseline).

	Model 1^a^ (*N* = 959)			Model 2^b^ (*N* = 946)			Model 3^c^ (*N* = 945)			Model 4^d^ (*N* = 945)		
Plasma GDF‐15	OR	95% CI	*p*	*p*	95% CI	*p*	OR	95% CI	*p*	OR	95% CI	*p*
Threshold 1: Cut‐off												
Low GDF‐15 (< 1500 pg/mL)	Ref.			Ref.			Ref.					
High GDF‐15 (≥ 1500 pg/mL)	**3.63**	**1.61–8.20**	**0.002**	**3.57**	**1.58–8.05**	**0.002**	**3.56**	**1.58–8.03**	**0.002**	**3.49**	**1.46–8.32**	**0.005**
Threshold 2: Quartiles												
GDF‐15 Q1 (≤ 799 pg/mL)	Ref.			Ref.			Ref.					
GDF‐15 Q2 (> 799.0, ≤ 1009.0 pg/mL)	3.10	0.32–30.10	0.330	3.05	0.31–29.67	0.337	3.04	0.31–29.63	0.338	1.87	0.18–19.36	0.598
GDF‐15 Q3 (> 1009.0, ≤ 1312.0 pg/mL)	**8.96**	**1.11–72.22**	**0.039**	**8.85**	**1.10–71.52**	**0.041**	**8.87**	**1.10–71.74**	**0.041**	**8.67**	**1.05–71.77**	**0.045**
GDF‐15 Q4 (> 1312.0 pg/mL)	**20.44**	**2.60–160.34**	**0.004**	**20.06**	**2.55–157.75**	**0.004**	**20.25**	**2.57–159.56**	**0.004**	**19.98**	**2.46–162.6**	**0.005**
GDF‐15 as continuous												
1‐SD increase	**1.60**	**1.25–2.04**	**<0.001**	**1.58**	**1.24–2.01**	**<0.001**	**1.58**	**1.24–2.01**	**<0.001**	**1.64**	**1.26–2.15**	**<0.001**

*Note:* Significant associations in bold.

Abbreviations: CI, confidence interval; GDF‐15, growth differentiation factor 15; OR, odds ratio of increasing frailty severity over time compared with the reference group.

^a^ Adjusted by age and sex only.

^b^ Adjusted by age, sex, body mass index, MAPT group and education.

^c^ Adjusted by age, sex, body mass index, MAPT group, education and plasma IL6.

^d^ Adjusted by age, sex, body mass index, MAPT group, education, plasma IL6 and number of comorbidities.

### 3.3 Cross‐Sectional Stratified Analyses by Sex and Age

The results of the exploratory sex‐stratified analyses suggest an overall stronger association between GDF‐15 levels and frailty prevalence among women compared with male sex participants, despite some quartile‐based analyses not being possible due to reduction of statistical power. Age‐stratified analyses did not show remarkable differences among individuals with ≤ 75 or > 75 years of age (Tables S3–S8).

### 3.4 Longitudinal Associations With Frailty Onset and Progression

In longitudinal analyses, baseline GDF‐15 was not associated with the evolution in the number of frailty criteria in the mixed‐effects logistic regression models (Table 4), but we found a greater risk of presenting frailty (≥ 3 frailty criteria) among those prefrail/robust at baseline (*n* = 865, Model 1, hazard ratio, HR = 1.69, 1.03–2.78, *p* = 0.039) with high GDF‐15 levels, when characterized based on the ≥ 1500 pg/mL cut‐point. This association faded in the full‐adjusted Cox regression model (*p* = 0.057, Table 5).

**TABLE 4:** Mixed‐effect ordinal logistic regression examining associations between plasma GDF‐15 and frailty evolution over time.

Plasma GDF‐15	Model 1^a^ (*N* = 1085)			Model 2^b^ (*N* = 1064)			Model 3^c^ (*N* = 1063)			Model 4^d^ (*N* = 1061)		
	OR	95% CI	*p*	*p*	95% CI	*p*	OR	95% CI	*p*	OR	95% CI	*p*
Threshold 1: cut‐off												
Low GDF‐15 (< 1500 pg/mL)	Ref.			Ref.			Ref.					
High GDF‐15 (≥ 1500 pg/mL)	1.07	1.98–1.15	0.120	1.06	0.98–1.14	0.173	1.06	0.97–1.14	0.167	1.05	0.97–1.14	0.203
Threshold 2: Quartiles												
GDF‐15 Q1 (≤ 799 pg/mL)	Ref.											
GDF‐15 Q2 (> 799.0, ≤ 1009.0 pg/mL)	0.97	0.91–1.05	0.480	0.97	0.90–1.05	0.464	0.97	0.91–1.05	0.488	0.98	0.91–1.05	0.570
GDF‐15 Q3 (> 1009.0, ≤ 1312.0 pg/mL)	0.96	0.88–1.02	0.229	0.95	0.89–1.03	0.215	0.96	0.89–1.03	0.230	0.96	0.89–1.03	0.282
GDF‐15 Q4 (> 1312.0 pg/mL)	1.01	0.94–1.09	0.815	0.99	0.93–1.07	0.961	1.00	0.93–1.08	0.988	1.00	0.93–1.08	0.919
GDF‐15 as continuous												
1‐SD increase	1.02	0.99–05	0.200	1.01	0.99–1.04	0.294	1.02	0.99–1.04	0.277	1.02	0.99–1.04	0.272

Abbreviations: CI, confidence interval; GDF‐15, growth differentiation factor 15; OR, odds ratio of increasing frailty severity over time compared with the reference group.

^a^ Adjusted by age and sex only.

^b^ Adjusted by age, sex, body mass index, MAPT group and education.

^c^ Adjusted by age, sex, body mass index, MAPT group, education and plasma IL6.

^d^ Adjusted by age, sex, body mass index, MAPT group, education, plasma IL6 and number of comorbidities.

**TABLE 5:** Cox proportional hazard models for incident frailty over the follow‐up.

Plasma GDF‐15	Model 1^a^ (*N* = 865)			Model 2^b^ (*N* = 853)			Model 3^c^ (*N* = 852)			Model 4^d^ (*N* = 852)		
	HR	95% CI	*p*	HR	95% CI	*p*	HR	95% CI	*p*	HR	95% CI	*p*
Threshold 1: Cut‐off												
Low GDF‐15 (< 1500 pg/mL)	Ref.			Ref.								
High GDF‐15 (≥ 1500 pg/mL)	**1.69**	**1.03–2.78**	**0.039**	1.63	0.99–2.70	0.055	1.63	0.99–2.69	0.057	1.40	0.85–2.33	0.187
Threshold 2: Quartiles												
GDF‐15 Q1 (≤ 799 pg/mL)	Ref.			Ref.			Ref.					
GDF‐15 Q2 (> 799.0, ≤ 1009.0 pg/mL)	0.82	0.45–1.49	0.513	0.82	0.45–1.49	0.510	0.81	0.44–1.48	0.496	0.79	0.43–1.43	0.433
GDF‐15 Q3 (> 1009.0, ≤ 1312.0 pg/mL)	0.74	0.40–1.35	0.320	0.73	0.40–1.35	0.320	0.73	0.40–1.34	0.308	0.73	0.40–1.35	0.317
GDF‐15 Q4 (> 1312.0 pg/mL)	1.21	0.68–2.17	0.522	1.17	0.65–2.10	0.604	1.15	0.64–2.08	0.742	1.11	0.61–2.00	0.737
GDF‐15 as continuous												
1‐SD increase	1.15	0.97–1.36	0.120	1.14	0.96–1.35	0.143	1.13	0.95–1.34	0.155	1.10	0.92–1.32	0.285

Abbreviations: CI, confidence interval; GDF‐15, growth differentiation factor 15; HR, hazard ratio of increasing risk of frailty onset over time compared with the reference group.

^a^ Adjusted by age and sex only.

^b^ Adjusted by age, sex, body mass index, MAPT group and education.

^c^ Adjusted by age, sex, body mass index, MAPT group, education and plasma IL6.

^d^ Adjusted by age, sex, body mass index, MAPT group, education, plasma IL6 and number of comorbidities.

### 3.5 Longitudinal Stratified Analyses by Sex and Age

Sex‐ and age‐stratified analyses did not show differences among groups, except for a greater risk of incident frailty (when GDF‐15 was characterized both as continuous and based on Groarke's cut‐point) when high versus low GDF‐15 was used as the exposure among younger individuals (Tables S9–S14).

### 3.6 Sensitivity Analyses

Sensitivity analyses including other inflammatory biomarkers as confounders showed virtually the same results (Tables S15–S20).

## 4 Discussion

Our results showed that higher levels of GDF‐15 were cross‐sectionally associated with a higher number of frailty criteria and the odds of presenting frailty in a relatively big sample of healthy community‐dwelling older adults. However, longitudinal analyses showed a low ability of plasma GDF‐15 to prognosticate evolution in the number of present frailty criteria. According to our results, very high levels of GDF‐15 at baseline might associate with increased risk of developing frailty along 4‐years of follow‐up when adjusted for age and sex; however, this association disappeared when further adjustment was added. Collectively, these results suggest a potential role of high plasma GDF‐15 as a biomarker of prevalent frailty, with a modest long‐term prognostic value.

This is consistent with an accumulating number of studies showing associations between higher GDF‐15 activity (expression, circulating levels) and physical and mental capacities [17, 33], functional ability [30], sarcopenia [31] and cachexia [34, 35] at older age, surrogates of the endpoint here investigated. Importantly, in the realms of frailty, the number of studies is scarce, and most of these included severely diseased populations [24, 25, 26] or were performed with small samples of older adults [36]. Therefore, existing evidence might be limitedly informative on the role of GDF‐15 as an early marker of frailty in the context of the ‘normal’ ageing process. Our results address this evidence scarcity by exploring the role of GDF‐15 in a relatively healthy population, showing significant associations restricted to the highest levels of GDF‐15, and pointing to the existence of a threshold below which no frailty heightened risk is evident, potentially indicating a physiological range at which GDF‐15 is not mediating the physiological changes associated with these conditions, but playing an adaptive role that might be even beneficial for health. These observations might support the putative pleiotropic nature of GDF‐15 [37].

To our knowledge, only one study has investigated the prospective associations of GDF‐15 and frailty incidence in an older adult sample so far. Oba et al. [36] showed an association between belonging to the highest serum GDF‐15 tertile (but not the middle tertile) and the risk of developing frailty (assessed by a modified version of the Frailty Phenotype and the Kihon Checklist) along a 3‐year follow‐up in a sample of older adults with cardiometabolic diseases (*n* = 17; mean age = 77 ± 6; 63% female sex). Similarly to this study, we also found a trend towards an association between very high plasma levels of GDF‐15 and frailty onset. However, direct comparability with our study might be limited due to disparities in the features of the sample (diseased vs. healthy), methods used to assess GDF‐15 (serum vs. plasma) and frailty (modified frailty phenotype and Kihon vs. frailty phenotype).

In the scope of the MAPT study, a previous work by Angioni et al. showed higher levels of plasma GDF‐15 among individuals with disease‐related frailty (but not in those with age‐related frailty) compared with nonfrail counterparts [38]. Also, Lengelé et al. [16] explored longitudinal associations between GDF‐15 and nutritional status and weight loss in MAPT, pointing to a role of GDF‐15 in the development of malnutrition [16], probably through reductions in appetite with ageing and disease [15]. However, none of these investigations examined the predictive associations of GDF‐15 with frailty incidence and worsening [4].

## 5 Strengths and Limitations

Our study presents strengths such as its large sample size, compared with previous studies, its longitudinal nature with a relatively long follow‐up and multiple time‐points of data collection, which allowed us to assess the evolution of the number of frailty criteria and the onset of frailty comprehensively. Our study is one of the first to explore GDF‐15‐frailty associations in a population of relatively healthy older adults. Our study has some limitations such as the homogeneity of the highly selected sample of RCT participants and the subsequent potential for sampling bias. Also, the figures of frailty incidence were reduced, which limits the statistical power. Additionally, GDF‐15 was assessed at the 1‐year visit, which limits causal inference. In the future, associations might be better captured by using samples from purely observational studies including diverse populations to overcome the limited generalizability of our results. Given the lack of a cut‐point for GDF‐15 for frailty in the literature, we chose that proposed by Groarke et al. Importantly, in their study, they chose this cut‐point to assure the inclusion of individuals with very high GDF‐15 levels within a pathological potential range, which explains our decision. However, the performance of this value across samples might differ according to the features of the population of interest, differences on the sensitivity of assessment kits, and the endpoint investigated, and therefore its adoption as an arbitrary marker of disease is discouraged. Finally, the ability of plasma GDF‐15 to reflect levels at the organ/cellular‐specific level is not fully established [25].

## 6 Conclusions

The present study found that higher plasma levels of GDF‐15 were cross‐sectionally associated with an increased prevalence of frailty in a community‐dwelling older adult population. Very high GDF‐15 levels at baseline were also linked to a slightly increased risk of developing frailty over a 4‐year follow‐up, although associations were limited to the greatest values and faded when confounders were considered, which indicates a modest ability to prognosticate the outcomes in the long term. These findings suggest that GDF‐15 could serve as a useful biomarker for identifying older adults with frailty. Future studies should examine if GDF‐15, and ideally the evolution in its levels, has a causative role in frailty in older adults without specific diseases.

## Funding

This study was supported by the Agence Nationale de la Recherche under the France 2030 program (reference number: ANR‐23‐IAHU‐0011). Additional funding was provided by the Gérontopôle of Toulouse, the French Ministry of Health (PHRC 2008 and PHRC 2009), the Pierre Fabre Research Institute (manufacturer of the omega‐3 supplement), ExonHit Therapeutics SA and Avid Radiopharmaceuticals Inc. Data‐sharing activities were supported by the Association Monegasque pour la Recherche sur la maladie d‘Alzheimer (AMPA) and the INSERM–University of Toulouse III UMR 1295 Unit. This work was conducted within the Inspire Program, a research platform supported by grants from the Region Occitanie/Pyrénées–Méditerranée (reference number: 1901175) and the European Regional Development Fund (ERDF; project number: MP0022856). The study also received funding from Alzheimer Prevention in Occitania and Catalonia (APOC Chair of Excellence—Inspire Program).

## Ethics Statement

The present work follows the ethical guidelines for authorship and publishing in the *Journal of Cachexia, Sarcopenia and Muscle* [39].

## Conflicts of Interest

The authors declare no conflicts of interest.

## Supporting information


**Table S1:** Baseline characteristics of the study population according by sex.
**Table S2:** Comparison of MAPT participants included/excluded in present analyses.
**Table S3:** Sex‐stratified ordinal logistic regression examining cross‐sectional associations between plasma GDF‐15 and frailty status (odds of presenting 1 frailty trait more at baseline).
**Table S4:** Sex‐stratified logistic regression examining cross‐sectional associations between plasma GDF‐15 and frailty (presenting > 3 criteria at baseline).
**Table S5:** Age‐stratified ordinal logistic regression examining cross‐sectional associations between plasma GDF‐15 and frailty status (odds of presenting 1 frailty trait more at baseline).
**Table S6:** Age‐stratified logistic regression examining cross‐sectional associations between plasma GDF‐15 and frailty (presenting > 3 criteria at baseline).
**Table S7:** Sex‐ group stratified mixed‐effect ordinal logistic regression examining associations between plasma GDF‐15 and frailty evolution over time.
**Table S8:** Sex‐stratified Cox proportional hazard models for incident frailty over the follow‐up.
**Table S9:** Age‐group stratified mixed‐effect ordinal logistic regression examining associations between plasma GDF‐15 and frailty evolution over time.
**Table S10:** Age‐stratified Cox proportional hazard models for incident frailty over the follow‐up.
**Table S11:** Ordinal logistic regression examining cross‐sectional associations between plasma GDF‐15 and frailty status (odds of presenting 1 frailty trait more at baseline).
**Table S12:** Logistic regression examining cross‐sectional associations between plasma GDF‐15 and frailty (presenting > 3 criteria at baseline).
**Table S13:** Mixed‐effect ordinal logistic regression examining associations between plasma GDF‐15 and frailty evolution over time.
**Table S14:** Cox proportional hazard models for incident frailty over the follow‐up.

## Data Availability

The data that support the findings of this study are available from MAPT Study Group but restrictions apply to the availability of these data, which were used under licence for the current study and so are not publicly available. Data are however available from the authors upon reasonable request and with permission of Nicola Coley (nicola.coley@inserm.fr).
