# Supplementary material for: Associations Between Growth Differentiating Factor‐15 and Frailty in Older Adults From the MAPT Study
Source: J Cachexia Sarcopenia Muscle. 2026 Jan 20;17(1):e70182. doi: 10.1002/jcsm.70182 (PMC12820345; doi:10.1002/jcsm.70182)
Supplement: Supplementary file 1 — Table S1: Baseline characteristics of the study population according by sex. Table S2: Comparison of MAPT participants included/excluded in present analyses. Table S3: Sex‐stratified ordinal logistic regression examining cross‐sectional associations between plasma GDF‐15 and frailty status (odds of presenting 1 frailty trait more at baseline). Table S4: Sex‐stratified logistic regression examining cross‐sectional associations between plasma GDF‐15 and frailty (presenting > 3 criteria at baseline). Table S5: Age‐stratified ordinal logistic regression examining cross‐sectional associations between plasma GDF‐15 and frailty status (odds of presenting 1 frailty trait more at baseline). Table S6: Age‐stratified logistic regression examining cross‐sectional associations between plasma GDF‐15 and frailty (presenting > 3 criteria at baseline). Table S7: Sex‐ group stratified mixed‐effect ordinal logistic regression examining associations between plasma GDF‐15 and frailty evolution over time. Table S8: Sex‐stratified Cox proportional hazard models for incident frailty over the follow‐up. Table S9: Age‐group stratified mixed‐effect ordinal logistic regression examining associations between plasma GDF‐15 and frailty evolution over time. Table S10: Age‐stratified Cox proportional hazard models for incident frailty over the follow‐up. Table S11: Ordinal logistic regression examining cross‐sectional associations between plasma GDF‐15 and frailty status (odds of presenting 1 frailty trait more at baseline). Table S12: Logistic regression examining cross‐sectional associations between plasma GDF‐15 and frailty (presenting > 3 criteria at baseline). Table S13: Mixed‐effect ordinal logistic regression examining associations between plasma GDF‐15 and frailty evolution over time. Table S14: Cox proportional hazard models for incident frailty over the follow‐up. [file JCSM-17-e70182-s001.docx]

**Supplementary Table 1. Baseline characteristics of the study population according by sex.**

| **Variables** | **Total** | **Women** | **Men** |
| --- | --- | --- | --- |
| **N (%)** | 1096 | 699 (63.8%) | 397 (36.2%) |
| **Age** | 75.3±4.4 | 75.3±4.3 | 75.4±4.5 |
| **MAPT Group** |  |  |  |
| Multidomain intervention + Omega 3 | 274 (25%) | 162 (23.18%) | 112 (28.2%) |
| Omega-3 | 267 (24.4%) | 173 (24.75%) | 94 (23.7%) |
| Multidomain intervention | 276 (25.2%) | 178 (25.46%) | 98 (24.7%) |
| Placebo | 279 (25.5%) | 186 (26.6%) | 93 (23.4%) |
| **Body Mass Index** (kg/m^2^) | 26.2±0.12 | 25.9±4.3 | 26.7±3.5*** |
| **Education** |  |  |  |
| No diploma or primary school certificate | 218 (21.0%) | 158 (22.9%) | 70 (18.0%) |
| Secondary education | 354 (32.8%) | 236 (34.3%) | 118 (30.3%) |
| High school diploma | 168 (15.6%) | 107 (15.5%) | 61 (15.6%) |
| University level | 329 (30.5%) | 188 (27.3%) | 141 (36.1%) |
| **Fried Frailty Phenotype** |  |  |  |
| Robust | 507 (52.9%) | 314 (51.2%) | 193 (55.8%) |
| Pre-Frail | 420 (43.8%) | 277 (45.2%) | 143 (41.3%) |
| Frail | 32 (3.3%) | 22 (3.6%) | 10 (2.9%) |
| **WL** | 41 (4.7%) | 37 (5.4%) | 14 (3.6%) |
| **GDF-15 (pg/mL)** | 1127.4±15.5 | 1041.1±483.1 | 1279.3±525.8*** |
| **IL-6 (pg/mL)** | 3.89±12.2 | 3.73±14.11 | 4.18±7.75 |

GDF-15, Growth Differentiation Factor 15; IL-6: interleukin 6;

**Supplementary Table 2. Comparison of MAPT participants included/excluded in present analyses**

| **Variables** | **Total** | **Excluded** | **Included** |
| --- | --- | --- | --- |
| **N (%)** | 1,679 | 583 (34.72) | 1,096 (65.28) |
| **Age** | 75.34±4.37 | 75.40±4.52 | 75.30±4.37 |
| **Female Sex** | 613 (63.92%) | 553 (67.77%) | 60 (41.96%)*** |
| **MAPT Group** |  |  |  |
| Multidomain intervention + Omega 3 | 417 (24.84%) | 143 (24.53%) | 274 (25.00%) |
| Omega-3 | 422 (25.13%) | 155 (26.159%) | 267 (24.36%) |
| Multidomain intervention | 420 (25.01%) | 144 (24.70%) | 276 (25.18%) |
| Placebo | 420 (25.01%) | 141 (24.19%) | 279 (25.46%) |
| **Body Mass Index** (kg/m^2^) | 26.27±4.04 | 26.47±3.98 | 26.22±4.05 |
| **Education** |  |  |  |
| No diploma | 85 (5.17%) | 36 (6.38%) | 49 (4.54%) |
| Primary school certificate | 286 (17.41%) | 107 (28.97%) | 179 (15.59%) |
| Secondary education | 553 (33.66%) | 199 (35.28%) | 354 (32.81%) |
| High school diploma | 242 (14.73%) | 74 (13.12%) | 168 (15.57%) |
| University level | 477 (29.03%) | 148 (26.24%) | 329 (30.49%) |

**Supplementary Table 3. Sex-stratified ordinal logistic regression examining cross-sectional associations between plasma GDF-15 and frailty status (odds of presenting 1 frailty trait more at baseline)**

| **Plasma GDF-15** | **Men^‡^**  **(N=341)** | | | **Women^‡^**  **(N=604)** | | |
| --- | --- | --- | --- | --- | --- | --- |
| **Threshold 1: cutoff** | **OR** | **95%CI** | **p-value** | **OR** | **95%CI** | **p-value** |
| Low GDF-15 (<1500 pg/mL) | **Ref.** | | | **Ref.** | | |
| High GDF-15 (≥1500 pg/mL) | **1.81** | **1.10-2.99** | **0.020** | **1.71** | **1.01-2.93** | **0.048** |
| **Threshold 2: quartiles** |  |  |  |  |  |  |
| GDF-15 Q1 (≤799 pg/mL) | **Ref.** | | | **Ref.** | | |
| GDF-15 Q2 (>799.0, ≤1009.0 pg/mL) | 1.26 | 0.67-2.39 | 0.467 | 0.92 | 0.58-1.45 | 0.712 |
| GDF-15 Q3 (>1009.0, ≤1312.0 pg/mL) | 1.23 | 0.64-2.34 | 0.529 | 0.95 | 0.60-1.51 | 0.838 |
| GDF-15 Q4 (>1312.0 pg/mL) | **2.36** | **1.20-4.61** | **0.013** | **1.64** | **1.01-2.66** | **0.046** |
| **GDF-15 as continuous** |  | | |  | | |
| 1-SD increase | **1.28** | **1.01-1.63** | **0.038** | 1.19 | 0.99-1.44 | 0.069 |

OR, hazard ratio of increasing risk of frailty onset over time compared to reference group

GDF-15, Growth Differentiation Factor 15; CI, confidence interval

Adjusted by age, body mass index, MAPT group, education, comorbidities, and plasma IL6.

**Supplementary Table 4. Sex-stratified logistic regression examining cross-sectional associations between plasma GDF-15 and frailty (presenting > 3 criteria at baseline)**

| **Plasma GDF-15** | **Men^‡^**  **(N=341)** | | | **Women^‡^**  **(N=604)** | | |
| --- | --- | --- | --- | --- | --- | --- |
| **Threshold 1: cutoff** | **OR** | **95%CI** | **p-value** | **OR** | **95%CI** | **p-value** |
| Low GDF-15 (<1500 pg/mL) | **Ref.** | | | **Ref.** | | |
| High GDF-15 (≥1500 pg/mL) | 2.18 | 0.51-9.35 | 0.291 | **4.82** | **1.83-12.67** | **0.001** |
| **Threshold 2: quartiles** |  |  |  |  |  |  |
| GDF-15 Q1 (≤799 pg/mL) | **Ref.** | | | **Ref.** | | |
| GDF-15 Q2 (>799.0, ≤1009.0 pg/mL) | - | - | - | 1.99 | 0.18-22.34 | 0.577 |
| GDF-15 Q3 (>1009.0, ≤1312.0 pg/mL) | - | - | - | 2.82 | 0.29-27.80 | 0.372 |
| GDF-15 Q4 (>1312.0 pg/mL) | **-** | **-** | **-** | **14.61** | **1.86-117.48** | **0.011** |
| **GDF-15 as continuous** |  | | |  | | |
| 1-SD increase | 1.65 | 0.95-2.87 | 0.076 | **1.58** | **1.20-2.09** | **0.001** |

OR, hazard ratio of increasing risk of frailty onset over time compared to reference group

GDF-15, Growth Differentiation Factor 15; CI, confidence interval

Adjusted by age, body mass index, MAPT group, education, comorbidities, and plasma IL6.

**Supplementary Table 5. Age-stratified ordinal logistic regression examining cross-sectional associations between plasma GDF-15 and frailty status (odds of presenting 1 frailty trait more at baseline)**

| **Plasma GDF-15** | **≤75 years old**  **(N=539)** | | | **>75 years old**  **(N=406)** | | |
| --- | --- | --- | --- | --- | --- | --- |
| **Threshold 1: cutoff** | **OR** | **95%CI** | **p-value** | **OR** | **95%CI** | **p-value** |
| Low GDF-15 (<1500 pg/mL) | **Ref.** | | | **Ref.** | | |
| High GDF-15 (≥1500 pg/mL) | **1.77** | **1.01-3.10** | **0.048** | **1.82** | **1.14-2.93** | **0.012** |
| **Threshold 2: quartiles** |  |  |  |  |  |  |
| GDF-15 Q1 (≤799 pg/mL) | **Ref.** | | | **Ref.** | | |
| GDF-15 Q2 (>799.0, ≤1009.0 pg/mL) | 0.91 | 0.54-1.51 | 0.702 | 0.87 | 0.50-1.50 | 0.613 |
| GDF-15 Q3 (>1009.0, ≤1312.0 pg/mL) | 1.05 | 0.63-1.74 | 0.847 | 1.02 | 0.58-1.77 | 0.952 |
| GDF-15 Q4 (>1312.0 pg/mL) | **2.04** | **1.21-3.45** | **0.007** | 1.53 | 0.84-2.79 | 0.164 |
| **GDF-15 as continuous** |  | | |  | | |
| 1-SD increase | 1.18 | 0.98-1.43 | 0.073 | **1.24** | **1.01-1.51** | **0.041** |

OR, hazard ratio of increasing risk of frailty onset over time compared to reference group

GDF-15, Growth Differentiation Factor 15; CI, confidence interval

Adjusted by sex, body mass index, MAPT group, education; comorbidities, and plasma IL6.

**Supplementary Table 6. Age-stratified logistic regression examining cross-sectional associations between plasma GDF-15 and frailty (presenting > 3 criteria at baseline)**

| **Plasma GDF-15** | **≤75 years old**  **(N=539)** | | | **>75 years old**  **(N=406)** | | |
| --- | --- | --- | --- | --- | --- | --- |
| **Threshold 1: cutoff** | **OR** | **95%CI** | **p-value** | **OR** | **95%CI** | **p-value** |
| Low GDF-15 (<1500 pg/mL) | **Ref.** | | | **Ref.** | | |
| High GDF-15 (≥1500 pg/mL) | 3.84 | 0.90-16.40 | 0.070 | **3.27** | **1.22-8.74** | **0.018** |
| **Threshold 2: quartiles** |  |  |  |  |  |  |
| GDF-15 Q1 (≤799 pg/mL) | - | - | - | - | - | - |
| GDF-15 Q2 (>799.0, ≤1009.0 pg/mL) | - | - | - | - | - | - |
| GDF-15 Q3 (>1009.0, ≤1312.0 pg/mL) | - | - | - | - | - | - |
| GDF-15 Q4 (>1312.0 pg/mL) | - | - | - | - | - | - |
| **GDF-15 as continuous** |  | | |  | | |
| 1-SD increase | 1.25 | 0.87-1.78 | 0.231 | **1.71** | **1.24-2.36** | **0.001** |

OR, hazard ratio of increasing risk of frailty onset over time compared to reference group

GDF-15, Growth Differentiation Factor 15; CI, confidence interval

Adjusted by sex, body mass index, MAPT group, education, comorbidities, and plasma IL6.

**Supplementary Table 7. Sex- group stratified mixed-effect ordinal logistic regression examining associations between plasma GDF-15 and frailty evolution over time**

| **Plasma GDF-15** | **Men**  **(N=384)** | | | **Women**  **(N=680)** | | |
| --- | --- | --- | --- | --- | --- | --- |
| **Threshold 1: cutoff** | **OR** | **95%CI** | **p-value** | **OR** | **95%CI** | **p-value** |
| Low GDF-15 (<1500 pg/mL) | **Ref.** | | | **Ref.** | | |
| High GDF-15 (≥1500 pg/mL) | 1.06 | 0.95-1.19 | 0.277 | 1.07 | 0.95-1.20 | 0.288 |
| **Threshold 2: quartiles** |  |  |  |  |  |  |
| GDF-15 Q1 (≤799 pg/mL) |  | | | **Ref.** | | |
| GDF-15 Q2 (>799.0, ≤1009.0 pg/mL) | 1.02 | 0.89-1.18 | 0.719 | 0.98 | 0.89-1.07 | 0.588 |
| GDF-15 Q3 (>1009.0, ≤1312.0 pg/mL) | 1.02 | 0.88-1.17 | 0.798 | 0.95 | 0.87-1.03 | 0.223 |
| GDF-15 Q4 (>1312.0 pg/mL) | 1.07 | 0.93-1.23 | 0.325 | 0.97 | 0.89-1.06 | 0.488 |
| **GDF-15 as continuous** |  | | |  | | |
| 1-SD increase | 1.03 | 0.99-1.09 | 0.166 | 1.01 | 0.97-1.04 | 0.698 |

OR, hazard ratio of increasing risk of frailty onset over time compared to reference group

GDF-15, Growth Differentiation Factor 15; CI, confidence interval

Adjusted by age, body mass index, MAPT group, education; comorbidities, and plasma IL6.

**Supplementary Table 8. Sex-stratified Cox proportional hazard models for incident frailty over the follow-up**

| **Plasma GDF-15** | **Men^‡^**  **(N=305)** | | | **Women^‡^**  **(N=547)** | | |
| --- | --- | --- | --- | --- | --- | --- |
| **Threshold 1: cutoff** | **HR** | **95%CI** | **p-value** | **HR** | **95%CI** | **p-value** |
| Low GDF-15 (<1500 pg/mL) | **Ref.** | | | **Ref.** | | |
| High GDF-15 (≥1500 pg/mL) | 2.19 | 0.98-4.85 | 0.055 | 1.56 | 0.76-3.20 | 0.226 |
| **Threshold 2: quartiles** |  |  |  |  |  |  |
| GDF-15 Q1 (≤799 pg/mL) | **Ref.** | | | **Ref.** | | |
| GDF-15 Q2 (>799.0, ≤1009.0 pg/mL) | 2.38 | 0.63-9.04 | 0.203 | 0.98 | 0.49-1.97 | 0.964 |
| GDF-15 Q3 (>1009.0, ≤1312.0 pg/mL) | 1.35 | 0.36-5.08 | 0.654 | 0.65 | 0.30-1.40 | 0.273 |
| GDF-15 Q4 (>1312.0 pg/mL) | 3.56 | 0.99-12.83 | 0.053 | 1.14 | 0.56-2.28 | 0.721 |
| **GDF-15 as continuous** |  | | |  | | |
| 1-SD increase | 1.33 | 0.98-1.80 | 0.063 | 1.07 | 0.84-1.36 | 0.582 |

OR, hazard ratio of increasing risk of frailty onset over time compared to reference group

GDF-15, Growth Differentiation Factor 15; CI, confidence interval

Adjusted by age, body mass index, MAPT group, education; comorbidities, and plasma IL6.

**Supplementary Table 9. Age-group stratified mixed-effect ordinal logistic regression examining associations between plasma GDF-15 and frailty evolution over time**

| **Plasma GDF-15** | **≤75 years old**  **(N=609)** | | | **>75 years old**  **(N=454)** | | |
| --- | --- | --- | --- | --- | --- | --- |
| **Threshold 1: cutoff** | **OR** | **95%CI** | **p-value** | **OR** | **95%CI** | **p-value** |
| Low GDF-15 (<1500 pg/mL) | **Ref.** | | | **Ref.** | | |
| High GDF-15 (≥1500 pg/mL) | 1.06 | 0.94-1.20 | 0.332 | 1.03 | 0.80-3.87 | 0.154 |
| **Threshold 2: quartiles** |  |  |  |  |  |  |
| GDF-15 Q1 (≤799 pg/mL) | **Ref.** | | | **Ref.** | | |
| GDF-15 Q2 (>799.0, ≤1009.0 pg/mL) | 1.01 | 0.92-1.12 | 0.829 | 0.96 | 0.89-1.07 | 0.477 |
| GDF-15 Q3 (>1009.0, ≤1312.0 pg/mL) | 0.98 | 0.88-1.08 | 0.670 | 0.96 | 0.86-1.07 | 0.431 |
| GDF-15 Q4 (>1312.0 pg/mL) | 0.97 | 0.87-1.07 | 0.487 | 0.99 | 0.88-1.11 | 0.873 |
| **GDF-15 as continuous** |  | | |  | | |
| 1-SD increase | 1.01 | 0.98-1.05 | 0.555 | 1.00 | 0.96-1.04 | 0.593 |

OR, hazard ratio of increasing risk of frailty onset over time compared to reference group

GDF-15, Growth Differentiation Factor 15; CI, confidence interval

Adjusted by age, sex, body mass index, MAPT group, education, comorbidities, and plasma IL6.

**Supplementary Table 10. Age-stratified Cox proportional hazard models for incident frailty over the follow-up**

| **Plasma GDF-15** | **≤75 years old**  **(N=500)** | | | **>75 years old**  **(N=352)** | | |
| --- | --- | --- | --- | --- | --- | --- |
| **Threshold 1: cutoff** | **HR** | **95%CI** | **p-value** | **HR** | **95%CI** | **p-value** |
| Low GDF-15 (<1500 pg/mL) | **Ref.** | | | **Ref.** | | |
| High GDF-15 (≥1500 pg/mL) | **4.28** | **1.85-9.93** | **0.001** | 1.14 | 0.61-2.14 | 0.675 |
| **Threshold 2: quartiles** |  |  |  |  |  |  |
| GDF-15 Q1 (≤799 pg/mL) |  | | |  | | |
| GDF-15 Q2 (>799.0, ≤1009.0 pg/mL) | 1.17 | 0.46-2.98 | 0.746 | 1.06 | 0.54-2.05 | 0.873 |
| GDF-15 Q3 (>1009.0, ≤1312.0 pg/mL) | 0.57 | 0.18-1.77 | 0.333 | 0.71 | 0.34-1.47 | 0.355 |
| GDF-15 Q4 (>1312.0 pg/mL) | 1.88 | 0.75-4.74 | 0.179 | 1.04 | 0.49-2.18 | 0.923 |
| **GDF-15 as continuous** |  | | |  | | |
| 1-SD increase | **1.29** | **1.05-1.59** | **0.017** | 1.04 | 0.81-1.33 | 0.755 |

OR, hazard ratio of increasing risk of frailty onset over time compared to reference group

GDF-15, Growth Differentiation Factor 15; CI, confidence interval

Adjusted by sex, body mass index, MAPT group, education, comorbidities, and plasma IL6.

**Supplementary Table 11. Ordinal logistic regression examining cross-sectional associations between plasma GDF-15 and frailty status (odds of presenting 1 frailty trait more at baseline)**

| **Plasma GDF-15** | **TNFR1^¥^**  **(N=945)** | | | **MCP-1**  **(N=945)** | | | **CRP^¥^**  **(N=945)** | | |
| --- | --- | --- | --- | --- | --- | --- | --- | --- | --- |
| **Threshold 1: cutoff** | **OR** | **95%CI** | **p-value** | **OR** | **95%CI** | **p-value** | **OR** | **95%CI** | **p-value** |
| Low GDF-15 (<1500 pg/mL) | **Ref.** | | | **Ref.** | | | **Ref.** | | |
| High GDF-15 (≥1500 pg/mL) | **1.54** | **1.02-2.32** | **0.039** | **1.86** | **1.29-2.68** | **0.001** | **1.79** | **1.25-2.58** | **0.001** |
| **Threshold 2: quartiles** |  |  |  |  |  |  |  |  |  |
| GDF-15 Q1 (≤799 pg/mL) |  | | |  | | |  |  |  |
| GDF-15 Q2 (>799.0, ≤1009.0 pg/mL) | 0.99 | 0.68-1.45 | 0.960 | 1.08 | 0.74-1.57 | 0.688 | 1.03 | 0.71-1.50 | 0.868 |
| GDF-15 Q3 (>1009.0, ≤1312.0 pg/mL) | 1.10 | 0.74-1.64 | 0.634 | 1.27 | 0.86-1.85 | 0.226 | 1.20 | 0.82-1.74 | 0.353 |
| GDF-15 Q4 (>1312.0 pg/mL) | 1.46 | 0.90-2.36 | 0.124 | **1.94** | **1.28-2.95** | **0.002** | **1.76** | **1.18-2.63** | **0.005** |
| **GDF-15 as continuous** |  | | |  | | |  |  |  |
| 1-SD increase | **1.13** | **0.94-1.36** | **0.179** | **1.26** | **1.08-1.48** | **0.002** | **1.23** | **1.07-1.43** | **0.005** |

OR, odds ratio of increasing frailty severity over time compared to reference group

GDF-15, Growth Differentiation Factor 15; CI, confidence interval

**¥** : Adjusted by age, sex, body mass index, MAPT group, education, and the inflammatory marker.

**Supplementary Table 12. Logistic regression examining cross-sectional associations between plasma GDF-15 and frailty (presenting > 3 criteria at baseline)**

| **Plasma GDF-15** | **TNFR1^¥^**  **(N=945)** | | | **MCP-1**  **(N=945)** | | | **CRP^¥^**  **(N=945)** | | |
| --- | --- | --- | --- | --- | --- | --- | --- | --- | --- |
| **Threshold 1: cutoff** | **OR** | **95%CI** | **p-value** | **OR** | **95%CI** | **p-value** | **OR** | **95%CI** | **p-value** |
| Low GDF-15 (<1500 pg/mL) | **Ref.** | | | **Ref.** | | | **Ref.** | | |
| High GDF-15 (≥1500 pg/mL) | 2.23 | 0.85-5.91 | 0.104 | **3.45** | **1.51-7.90** | **0.003** | **3.56** | **1.58-8.06** | **0.002** |
| **Threshold 2: quartiles** |  |  |  |  |  |  |  |  |  |
| GDF-15 Q1 (≤799 pg/mL) |  | | |  | | |  |  |  |
| GDF-15 Q2 (>799.0, ≤1009.0 pg/mL) | 2.86 | 0.29-27.9 | 0.366 | 3.08 | 0.32-30.14 | 0.333 | 3.09 | 0.32-30.11 | 0.331 |
| GDF-15 Q3 (>1009.0, ≤1312.0 pg/mL) | 7.86 | 0.95-64.7 | 0.055 | 8.98 | 1.10-73.04 | 0.040 | 8.92 | 1.10-72.13 | 0.040 |
| GDF-15 Q4 (>1312.0 pg/mL) | **15.48** | **1.79-133.5** | **0.013** | **20.57** | **2.56-165.03** | **0.004** | **20.10** | **2.56-158.03** | **0.004** |
| **GDF-15 as continuous** |  | | |  | | |  |  |  |
| 1-SD increase | **1.39** | **1.01-1.91** | **0.043** | **1.57** | **1.22-2.01** | **<0.001** | **1.63** | **1.26-2.11** | **<0.001** |

OR, odds ratio of increasing frailty severity over time compared to reference group

GDF-15, Growth Differentiation Factor 15; CI, confidence interval

**¥** : Adjusted by age, sex, body mass index, MAPT group, education, and plasma IL6. **£** Adjusted by age, sex, body mass index, MAPT group, education, plasma IL6 and number of comorbidities

**Supplementary Table 13. Mixed-effect ordinal logistic regression examining associations between plasma GDF-15 and frailty evolution over time**

| **Plasma GDF-15** | **TNFR1^¥^**  **(N=945)** | | | **MCP-1**  **(N=945)** | | | **CRP^¥^**  **(N=945)** | | |
| --- | --- | --- | --- | --- | --- | --- | --- | --- | --- |
| **Threshold 1: cutoff** | **OR** | **95%CI** | **p-value** | **OR** | **95%CI** | **p-value** | **OR** | **95%CI** | **p-value** |
| Low GDF-15 (<1500 pg/mL) | **Ref.** | | | **Ref.** | | | **Ref.** | | |
| High GDF-15 (≥1500 pg/mL) | 1.06 | 0.98-1.14 | 0.167 | 1.06 | 0.98-1.14 | 0.172 | 1.05 | 0.98-1.14 | 0.182 |
| **Threshold 2: quartiles** |  |  |  |  |  |  |  |  |  |
| GDF-15 Q1 (≤799 pg/mL) |  | | |  | | |  |  |  |
| GDF-15 Q2 (>799.0, ≤1009.0 pg/mL) | 0.97 | 0.90-1.05 | 0.466 | 0.97 | 0.90-1.05 | 0.464 | 0.97 | 0.90-1.05 | 0.459 |
| GDF-15 Q3 (>1009.0, ≤1312.0 pg/mL) | 0.96 | 0.89-1.03 | 0.218 | 0.95 | 0.89-1.03 | 0.215 | 0.96 | 0.89-1.03 | 0.223 |
| GDF-15 Q4 (>1312.0 pg/mL) | 0.99 | 0.93-1.08 | 0.979 | 0.99 | 0.93-1.07 | 0.961 | 1.00 | 0.93-1.07 | 0.942 |
| **GDF-15 as continuous** |  | | |  | | |  |  |  |
| 1-SD increase | 1.23 | 0.97-1.56 | 0.093 | 1.01 | 0.99-1.04 | 0.294 | 1.01 | 0.99-1.04 | 0.306 |

OR, odds ratio of increasing frailty severity over time compared to reference group

GDF-15, Growth Differentiation Factor 15; CI, confidence interval

**¥** : Adjusted by age, sex, body mass index, MAPT group, education, and plasma IL6. **£** Adjusted by age, sex, body mass index, MAPT group, education, plasma IL6 and number of comorbidities

**Supplementary Table 14. Cox proportional hazard models for incident frailty over the follow-up**

| **Plasma GDF-15** | **TNFR1^¥^**  **(N=945)** | | | **MCP-1**  **(N=945)** | | | **CRP^¥^**  **(N=945)** | | |
| --- | --- | --- | --- | --- | --- | --- | --- | --- | --- |
| **Threshold 1: cutoff** | **OR** | **95%CI** | **p-value** | **OR** | **95%CI** | **p-value** | **OR** | **95%CI** | **p-value** |
| Low GDF-15 (<1500 pg/mL) | **Ref.** | | | **Ref.** | | | **Ref.** | | |
| High GDF-15 (≥1500 pg/mL) | 1.69 | 0.96-2.97 | 0.070 | 1.61 | 0.97-2.67 | 0.067 | 1.54 | 0.93-2.55 | 0.094 |
| **Threshold 2: quartiles** |  |  |  |  |  |  |  |  |  |
| GDF-15 Q1 (≤799 pg/mL) |  | | |  | | |  |  |  |
| GDF-15 Q2 (>799.0, ≤1009.0 pg/mL) | 0.81 | 0.44-1.48 | 0.491 | 0.80 | 0.44-1.46 | 0.461 | 0.81 | 0.45-1.47 | 0.492 |
| GDF-15 Q3 (>1009.0, ≤1312.0 pg/mL) | 0.72 | 0.38-1.36 | 0.308 | 0.71 | 0.38-1.32 | 0.283 | 0.73 | 0.40-1.34 | 0.306 |
| GDF-15 Q4 (>1312.0 pg/mL) | 1.12 | 0.56-2.22 | 0.749 | 1.11 | 0.61-2.04 | 0.733 | 1.13 | 0.62-2.03 | 0.693 |
| **GDF-15 as continuous** |  | | |  | | |  |  |  |
| 1-SD increase | 1.14 | 1.12-1.21 | 0.189 | 1.13 | 0.94-1.34 | 0.187 | 1.08 | 0.91-1.27 | 0.366 |

OR, hazard ratio of increasing risk of frailty onset over time compared to reference group

GDF-15, Growth Differentiation Factor 15; CI, confidence interval

**¥** : Adjusted by age, sex, body mass index, MAPT group, education, and plasma IL6. **£** Adjusted by age, sex, body mass index, MAPT group, education, plasma IL6 and number of comorbidities
